# MS-275 (Entinostat) Promotes Radio-Sensitivity in PAX3-FOXO1 Rhabdomyosarcoma Cells

**DOI:** 10.3390/ijms221910671

**Published:** 2021-10-01

**Authors:** Matteo Cassandri, Silvia Pomella, Alessandra Rossetti, Francesco Petragnano, Luisa Milazzo, Francesca Vulcano, Simona Camero, Silvia Codenotti, Francesca Cicchetti, Roberto Maggio, Claudio Festuccia, Giovanni Luca Gravina, Alessandro Fanzani, Francesca Megiorni, Marialuigia Catanoso, Cinzia Marchese, Vincenzo Tombolini, Franco Locatelli, Rossella Rota, Francesco Marampon

**Affiliations:** 1 Department of Radiotherapy, Policlinico Umberto I, Sapienza University of Rome, 00161 Rome, Italy; matteo.cassandri@opbg.net (M.C.); vincenzo.tombolini@uniroma1.it (V.T.); 2 Department of Oncohematology, Bambino Gesù Children’s Hospital, IRCCS, 00146 Rome, Italy; silvia.pomella@opbg.net (S.P.); marialuigia.catanoso@opbg.net (M.C.); franco.locatelli@opbg.net (F.L.); 3 Department of Biotechnological and Applied Clinical Sciences, University of L’Aquila, 67100 L’Aquila, Italy; alessandra.rossetti93@gmail.com (A.R.); francesco.petragnano@gmail.com (F.P.); roberto.maggio@univaq.it (R.M.); claudio.festuccia@univaq.it (C.F.); giovanniluca.gravina@uniroma1.it (G.L.G.); 4 Department of Oncology and Molecular Medicine, Istituto Superiore di Sanità, Viale Regina Elena, 00161 Rome, Italy; luisa.milazzo@iss.it (L.M.); francesca.vulcano@iss.it (F.V.); 5 Department of Maternal and Child and Urological Sciences, Sapienza University of Rome, 00161 Rome, Italy; simona.camero@uniroma1.it; 6 Department of Molecular and Translational Medicine, Division of Biotechnology, University of Brescia, 25121 Brescia, Italy; s.codenotti002@unibs.it (S.C.); alessandro.fanzani@unibs.it (A.F.); 7 Policlinico Umberto I Hospital, Viale del Policlinico, 00161 Rome, Italy; cicchettifrancesca2@gmail.com; 8 Department of Experimental Medicine, Sapienza University of Rome, Viale Regina Elena 324, 00161 Rome, Italy; francesca.megiorni@uniroma1.it (F.M.); cinzia.marchese@uniroma1.it (C.M.); 9 Department of Gynecology/Obstetrics and Pediatrics, Sapienza University of Rome, 00161 Rome, Italy

**Keywords:** rhabdomyosarcoma, radiotherapy, MS-275, HDACs, pediatric cancers, soft tissue sarcoma, DNA damage

## Abstract

Rhabdomyosarcoma (RMS) is the most common soft tissue sarcoma of childhood. About 25% of RMS expresses fusion oncoproteins such as PAX3/PAX7-FOXO1 (fusion-positive, FP) while fusion-negative (FN)-RMS harbors RAS mutations. Radiotherapy (RT) plays a crucial role in local control but metastatic RMS is often radio-resistant. HDAC inhibitors (HDACi) radio-sensitize different cancer cells types. Thus, we evaluated MS-275 (Entinostat), a Class I and IV HDACi, in combination with RT on RMS cells in vitro and in vivo. MS-275 reversibly hampered cell survival in vitro in FN-RMS RD (RASmut) and irreversibly in FP-RMS RH30 cell lines down-regulating cyclin A, B, and D1, up-regulating p21 and p27 and reducing ERKs activity, and c-Myc expression in RD and PI3K/Akt/mTOR activity and N-Myc expression in RH30 cells. Further, MS-275 and RT combination reduced colony formation ability of RH30 cells. In both cell lines, co-treatment increased DNA damage repair inhibition and reactive oxygen species formation, down-regulated *NRF2*, *SOD*, *CAT* and *GPx4* anti-oxidant genes and improved RT ability to induce G2 growth arrest. MS-275 inhibited in vivo growth of RH30 cells and completely prevented the growth of RT-unresponsive RH30 xenografts when combined with radiation. Thus, MS-275 could be considered as a radio-sensitizing agent for the treatment of intrinsically radio-resistant PAX3-FOXO1 RMS.

## 1. Introduction

Rhabdomyosarcoma (RMS), a highly aggressive form of cancer deriving from mesenchymal cells failing to differentiate into skeletal muscle, represents the most frequent soft tissue sarcoma in children and adolescents [1]. The two major RMS histological subtypes are the ‘alveolar’ (ARMS) and ‘embryonal’ (ERMS) variants. ARMS is frequently characterized by the expression of the PAX3- or PAX7-FOXO1 oncogenic “fusion proteins” (fusion-positive, FP) and often has a dismal prognosis [1]. ERMS, the most frequent subtype, is devoid of any fusion gene (fusion-negative, FN) but has specific mutations of the RAS and RTK pathways [1]. Notably, both FN-RMS and FP-RMS are developmental-derived tumors and, thus, are characterized by an epigenetic imbalance that finally affects core gene regulatory architecture and sustains the transformed phenotype [2,3,4,5,6,7,8]. First-line treatment of RMS includes surgery to eradicate the primary tumor, radiation therapy (RT) and chemotherapy, with RT considered the key contributing factor to the improved results recently reported [9]. However, local control failure rates remain still high for high-risk patients suggesting the need for radiosensitizing strategies.

The acetylation of histones, nucleosomal proteins, which pack the DNA, structurally supporting chromosomes, is the most common epigenetic-related mechanism regulating gene expression [10]. The acetyltransferases (HATs) and histone deacetylases (HDACs) orchestrate acetylation by transferring or removing an acetyl group from acetyl CoA to the ε-amino group of the lysine residue, respectively, switching between gene transcription permissive and repressive chromatin structure [10]. Moreover, HATs and HDACs also regulate protein acetylation, affecting their activity, localization and stability [11]. Thus, HATs and HDACs have a critical role in regulating many cellular processes [10,11] and their aberrant activity and/or expression has been shown to promote the onset and progression of many diseases [12], including cancer [13,14]. Particularly, HDACs have been identified as critical targets for new anticancer therapies and several HDAC inhibitors (HDACi) selected and successfully tested in preclinical settings against several tumor types [15]. However, although HDACi clinically improved outcomes in patients with hematological malignancies, they failed in solid tumors [16]. This inefficiency might depend on the ability of tumor cells to activate chemoresistance mechanisms and/or on the lack of selectivity of the pan-HDACi tested [17]. Moreover, many clinical studies have been prematurely stopped often due to the dose-limiting toxicities of pan-HDACi [18]. Thus, selective HDACi able to target either a single HDAC isoform (isoform-selective HDACi) or several HDACs within a single class (class-selective HDACi), have emerged as ideal chemical tools to elucidate the individual functions of each HDAC, in order to perform combination-based therapeutic strategy and to obtain the best clinical result with the lowest possible toxicity [19,20]. In agreement, HDACi have been investigated in combination with RT in several types of cancers showing radiosensitizing potential in vitro and in preclinical models [21,22,23,24,25].

Class I and IV HDACs have been shown to be deregulated in RMS [3,4,6]. In in vitro and in vivo models of RMS several HDACi have been successfully preclinically tested, alone or in combination with chemotherapy (CHT) [26,27,28,29,30,31,32,33,34,35] or RT [36,37]. However, HDACi failed in treating RMS patients when used alone or in combination with CHT [38,39,40,41,42]. Recently, our group has elucidated the ability of two pan-HDACi to radiosensitize RMS cells in vitro and in vivo [36,37]. MS-275 (Entinostat), a potent class I and IV–selective HDACi [43], has been recently used in RMS xenograft models and in patients demonstrating modest antitumor activity alone and in combination with standard-of-care cytotoxic agents [33,34].

In this manuscript, we investigated the radiosensitizing potential of MS-275 in the treatment of the two most representative RMS cell lines, RD (FN-RMS) and RH30 (FP-RMS) [44], in vitro and in vivo. We show that MS-275 affected tumor cell survival inducing a non-apoptotic cell death and G1 cell cycle arrest in both cell lines even if the effects on RH30 are irreversible while RD cells recovered from the cell cycle arrest after the drug washout. Molecularly, MS-275 reduced cyclins expression and up-regulated cyclin-dependent kinase inhibitors p21 and p27 expression in RD and RH30 cells, decreasing ERK phosphorylation and c-Myc expression in RD, and AKT phosphorylation and N-Myc in RH30 cells.

MS-275 and RT as single treatments markedly affected the capacity of RH30 cells to form colonies and combined treatment strongly enhanced this effect. Despite the triggering of DNA damage, the reduction of Reactive Oxygen species (ROS) associated with down-regulation of antioxidant genes such as *NRF2*, *SOD*, *CAT* and *GPx4* and the increase of G2 cell cycle arrest in both cell lines by the combination vs single treatments, co-treatment in RD cells did not show any enhancement of the inhibitor effect on colony formation, which was hindered only by RT. Interestingly, whereas in vivo RT treatment partially affected RD xenografts growth, it had no effects on RH30 xenografts suggesting they were resistant to RT, as already reported [37]. Conversely, MS-275 significantly inhibited RH30 tumor growth with modest effects on RD tumors. Finally, MS-275 and RT combined treatment strongly prevented the growth of xenografted RH30 cells whereas showed only a partial inhibitory effect on RD xenografts. Altogether, these results suggest that MS-275 could have radiosensitizing properties on FP-RMS.

## 2. Results

### 2.1. In Vitro, MS-275 Induces Growth Arrest and Cell Death of Human FN-RMS and FP-RMS

**Figure 1:**
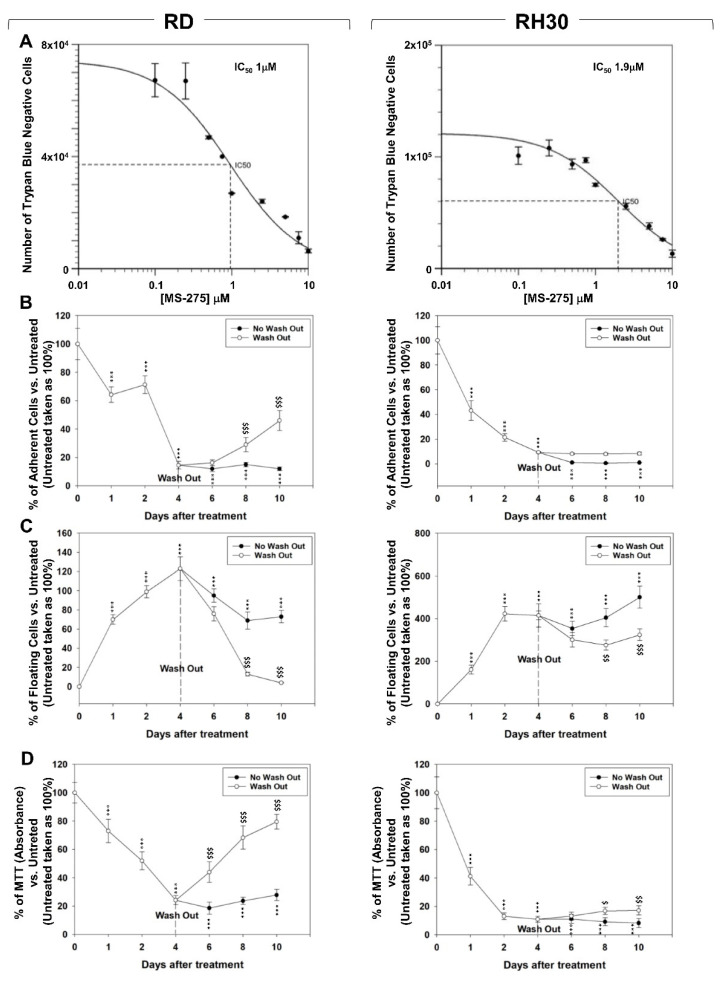
MS-275 induces reversible cell growth arrest in FN-RMS and FP-RMS cells. (**A**) Dose of MS-275 able to reduce by 50% the cell survival of RD (Left) and RH30 (Right) cell lines was identified treating the cells for 24 h with increasing concentrations of the drug. Cell viability was measured by Trypan Blue dye exclusion test. Results represent the mean values of three independent experiments ± SD. (**B**,**C**) Effect of MS-275 IC_50_ on cell number of adherent (**B**) and floating (**C**) RD (Left) and RH30 (Right) cells: after four days of treatment the drug was washed out or not and counts were performed for further 6 days. Surviving cells were counted using Trypan blue dye exclusion test. Results represent the mean values of four independent experiments ± SD. (**D**) Effect of MS-275 on cell viability, measured as metabolic activity by an MTT assay, of RD (Left) and RH30 (Right) cells treated as in (**B**,**C**). Results represent the mean values of four independent experiments ± SD. Statistical significance: *** *p* ≤ 0.001 vs. Untreated cells; ^$^ *p* ≤ 0.05, ^$$^ *p* ≤ 0.01, ^$$$^ *p* ≤ 0.001 Washout vs. No Washout.

**Figure 2:**
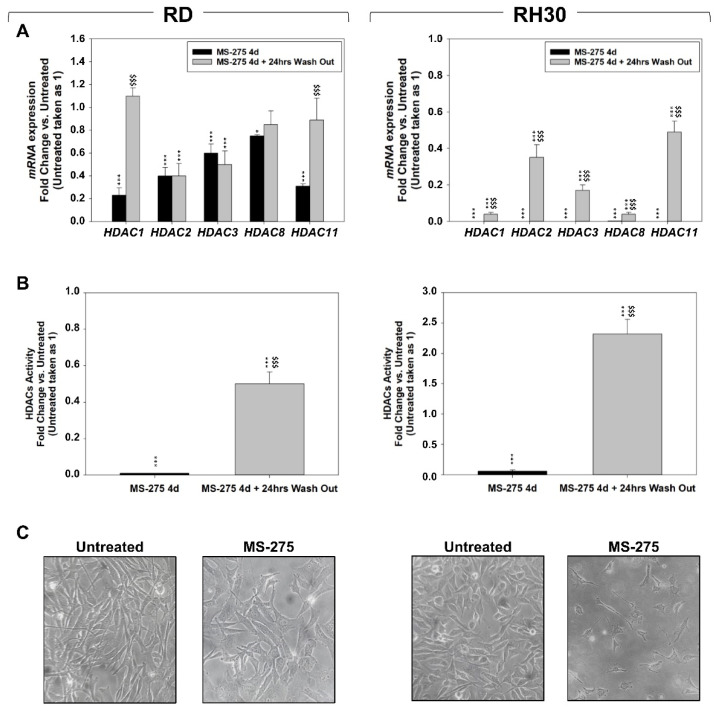
MS-275 reversibly reduces class I and IV HDACs expression and activity in FN-RMS and FP-RMS cells. (**A**) *HDAC1*, *2*, *3*, *8* and *11* transcript levels in RD (Left) and RH30 (Right) cells treated for 4 days with MS-275 (IC_50_) followed by 24 h of drug washout. Transcript levels were measured by qRT-PCR assays and *GAPDH* mRNA was used as endogenous control. The relative mRNA expression levels are presented as the average fold changes in treated tumor cell lines vs. untreated cells, set at 1. (**B**) HDACs activity in RD (Left) and RH30 (Right) cells treated as in (**A**) was measured by enzymatic assay and presented as the average fold changes. Results represent the mean values of three independent experiments ± SD. Statistical significance: * *p* ≤ 0.05, *** *p* ≤ 0.001 vs. Untreated cells; ^$$$^ *p* ≤ 0.001 Wash Out vs. No Wash Out. (**C**) Cellular morphology of RMS cells, RD (Left) and RH30 (Right), untreated or treated with MS-275 (IC_50_) for 4 days was analyzed under light microscope at 200X magnification.

The concentration of MS-275 able to inhibit the half of cell viability (IC_50_) at 24 h, assessed by Trypan Blue exclusion assay, was 1 μM in RD and 1.9 μM in RH30 cell lines (Figure 1A). These concentrations have been used in the experiments throughout the work. The effects of MS-275 on cell proliferation were assessed by counting adherent (Figure 1B) and floating (Figure 1C) RD and RH30 cells at different time points under 4 days of drug treatment followed or not by drug washout for a further 6 days. Four days of MS-275 treatment significantly reduced the number of adherent cells by 86.2 ± 3.4% in RD and 91.3 ± 4.3% in RH30 cells (Figure 1B) and, concomitantly, increased the number of floating cells (Figure 1C). Drug washout did not restore the growth potential of RH30 cells whereas RD cells slowly recovered (Figure 1B). The cellular metabolic activity was measured by MTT assay showing a reduction up to 4 days of MS-275 treatment and then, in agreement with the proliferation results in Figure 1B, an efficient or slight recovery in RD and RH30, respectively, after the drug washout (Figure 1D). After 4 days of MS-275, RD cells downregulated the transcript levels of *HDAC1* by ~77%, *HDAC2* ~60.5%, *HDAC3* ~40.9%, *HDAC8* ~25.9% and *HDAC11* ~69% (Figure 2A black columns, RD) as well as the global activity of HDACs by ~94.6% (Figure 2B black column, RD). Impressively, in RH30 cells the mRNA expression of all the investigated HDACs resulted totally repressed by the drug treatment (Figure 2A black columns, RH30) along with the global HDACs activity (Figure 2B black column, RH30). On the other hand, 24 h after the drug wash out the expression of transcript and activity levels were both efficiently restored and even up-regulated in the two cell lines (Figure 2A,B, gray columns). Notably, after 4 days of drug treatment, both RD and RH30 cells displayed the appearance of long cellular extensions, especially RH30 cells that exhibited a neuritic-like morphology (Figure 2C). Altogether, these findings indicate that MS-275 treatment resulted in both cytostatic and cytotoxic in the two RMS cell lines with a reversible on RD and irreversible on RH30 cells anti-proliferative effect.

### 2.2. MS-275 Affected Cell Cycle Distribution and Related Molecular Signature in RD and RH30 Cells

**Figure 3:**
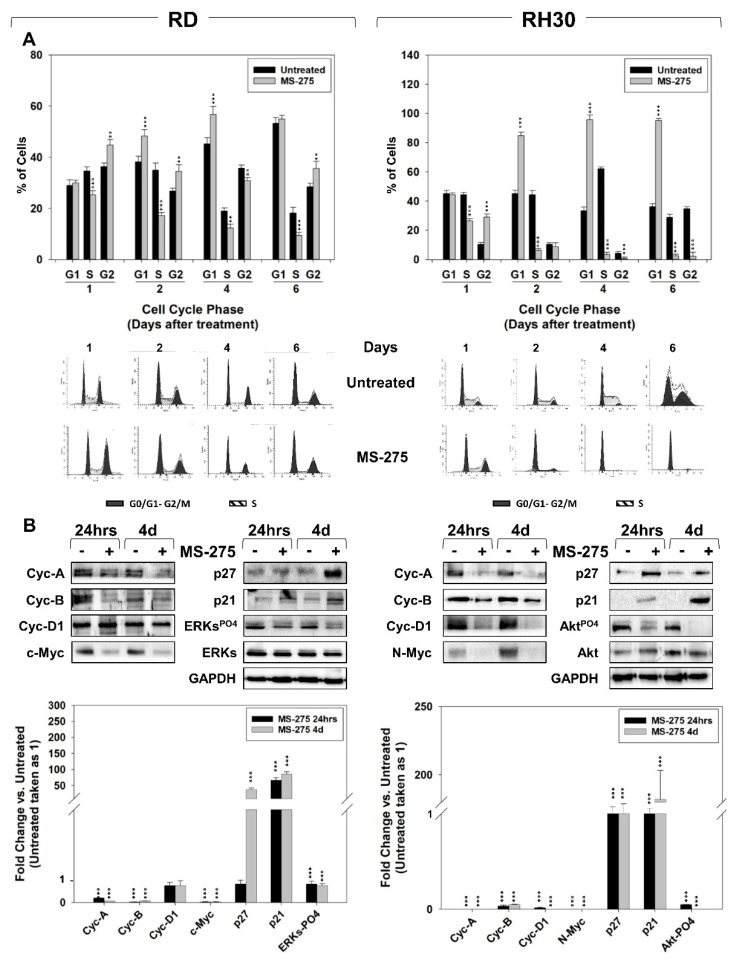
MS-275 affects cell cycle distribution, induces PARP1 but not caspase cell death cascades and promotes necrosis. (**A**) FACS analysis performed on RD (Left) and RH30 (Right) untreated or treated for 4 days with MS-275 (IC_50_). Data (Up) showing the percentage of cells in each cell cycle phase representing the mean value of three independent experiments. (Down) a representative of three independent experiments is shown. (**B**) Cell lysates from RD (Left) and RH30 (Right) cells treated for 24 h and 4 days with MS-275 (IC_50_) were analyzed by immunoblotting with specific antibodies for the indicated proteins; GAPDH expression was used as a loading control. Histograms of densitometric analysis (Down) represent the mean values of three independent experiments ± SD. Statistical significance: ** *p* ≤ 0.01, *** *p* ≤ 0.001 vs. Untreated cells.

We then investigated the effects of MS-275 on cell cycle distribution by performing flow cytometry on RD and RH30 cells at 1, 2, 4 and 6 days post-treatment. In both cell lines, MS-275 treatment resulted in a progressive reduction of the cell percentage in the S phase of the cell cycle vs. cells treated with the vehicle alone (hereafter indicated as untreated) (Figure 3A). Moreover, 6 days of MS-275 treatment increased the percentage of RD cells in the G2 phase that followed a transient accumulation in G1 at days 2 and 4 (Figure 3A, RD). Conversely, compared to untreated cells, MS-275-treated RH30 cells showed a marked accumulation in the G1 phase starting 2 days after treatment, which was further increased up to 6 days (Figure 3A, RH30). At the molecular level, a western blot analysis performed 24h and 4 days after treatment showed that, compared to untreated cells, both RD and RH30 cells surviving to MS-275 downregulated the expression of c-Myc in RD, N-Myc in RH30 and the cell cycle promoters cyclin-A (Cyc-A) and -B (Cyc-B), upregulating, in parallel, the expression of the cell cycle negative controllers p21 and p27 (Figure 3B). Cyclin-D1 (Cyc-D1) resulted also significantly downregulated by MS-275 in RH30 (Figure 3B, RH30) but not in RD (Figure 3B, RD). Notably, MS-275 reduced the activation status of ERKs and Akts in RD (Figure 3B, RD) and RH30 (Figure 3B, RH30) cells, respectively. Altogether, these evidences indicate that MS-275 differently modulates the cell cycle distribution in RD and RH30 cells even if it induces a similar modification in the expression of cell cycle molecular-related markers.

### 2.3. MS-275 Induced Non-Apoptotic Cell Death

The Annexin V cell staining on surviving cells was performed to characterize the cell death response to 24 h of MS-275.

**Figure 4:**
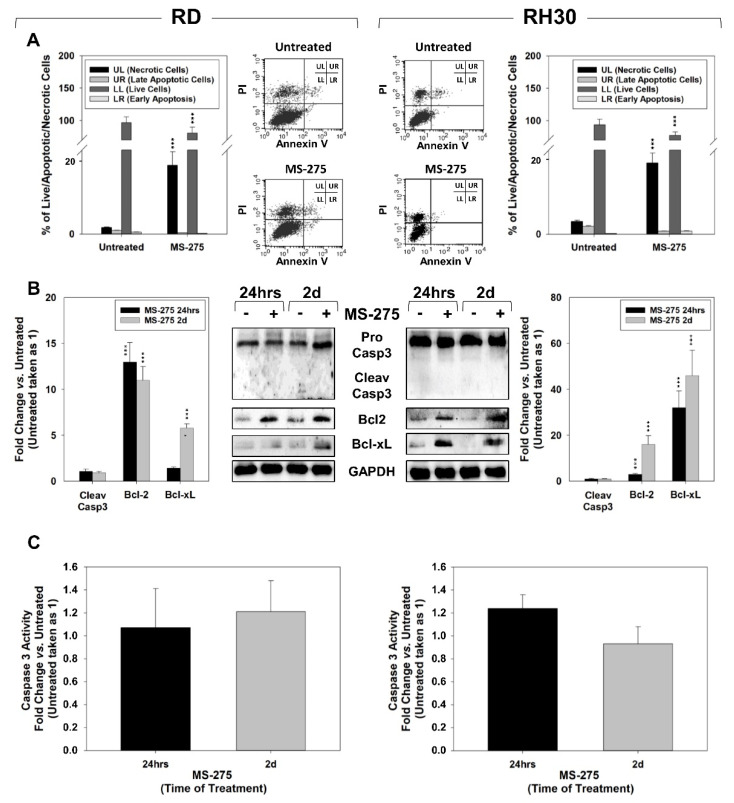
MS-275 induces PARP1 but not Caspase cell death cascades and promotes necrosis in FP-RMS cell lines. (**A**) Annexin V analysis performed on RD (Left) and RH30 (Right) cells untreated or treated for 24 h with MS-275 IC_50_. Histograms (RD left, RH30 right) showing the percentage of necrotic and apoptotic cells representing the mean value of three independent experiments. A FACS plot representative of three independent experiments is shown (RD left, RH30 right). (**B**) Cell lysates from RD (Left) and RH30 (Right) cells treated for 24 h and 2 days were analyzed by immunoblotting with specific antibodies for indicated proteins; GAPDH expression was used as a loading control. Histograms of densitometric analysis (RD left, RH30 right) represent the mean values of three independent experiments ± SD. (**C**) RD (Left) and RH30 (Right) cells treated as in (**B**) were analyzed for Caspase 3 activity by a specific assay. Histograms represent the mean values of three independent experiments ± SD. Statistical significance: *** *p* ≤ 0.001 vs. Untreated cells.

**Figure 5:**
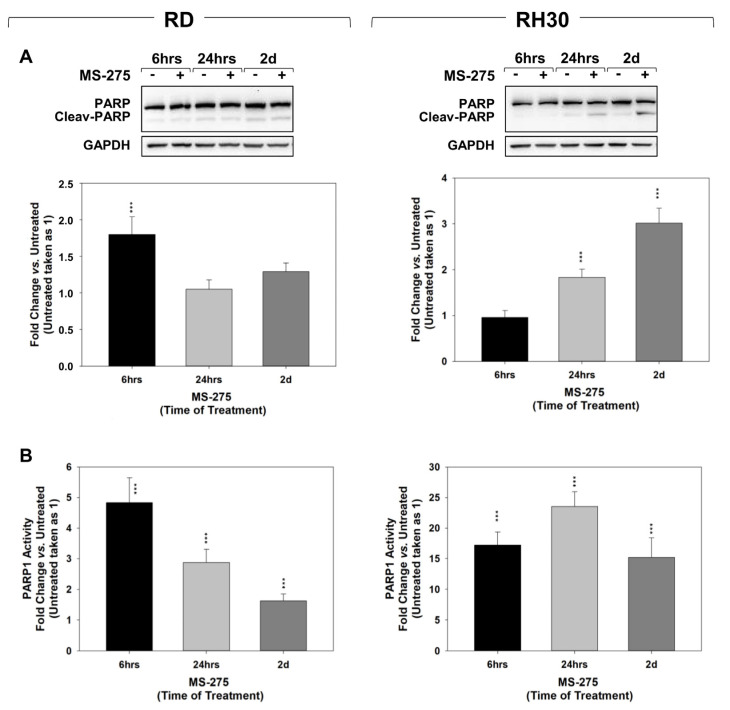
MS-275 induces PARP1 activation. (**A**) Cell lysates from RD (Left) and RH30 (Right) cells treated with MS-275 IC_50_ for 6 h, 24 h and 2 days were analyzed by immunoblotting with specific antibodies for the indicated proteins; GAPDH expression was used as loading control of samples. Histograms of densitometric analysis (RD left, RH30 right) represent the mean values of three independent experiments ± SD. (**B**) RD (Left) and RH30 (Right) cells treated as in (**A**) were analyzed for PARP1 activity by a specific assay. Histograms represent the mean values of three independent experiments ± SD. Statistical significance: *** *p* ≤ 0.001 vs. Untreated cells.

As shown in Figure 4A, MS-275 significantly increased the percentage of necrotic cells from ~1.8% to ~18% in RD and from ~4.4% to ~21% in RH30 cells compared to untreated cells, respectively. Conversely, no significant difference was seen in the early (LR) and late (UR) apoptotic populations (Figure 4A). In line with the lack of Annexin V positivity increase, 24 h and 2 days of MS-275 treatment did not up-regulate the expression (Figure 4B) and activity (Figure 4C) of the apoptosis-related marker cleaved-caspase 3, although it induced the expression of the anti-apoptotic Bcl-2 family proteins, Bcl-2 and Bcl-xL [45] (Figure 4B). Moreover, MS-275 increased the accumulation of cleaved PARP1 transiently in RD (Figure 5A, RD, 6 h) and later but stably in RH30 cells (Figure 5A, RH30, 24 h and 2 days). Interestingly, PARP1 activity resulted quickly and stably increased in both cell lines compared to untreated cells taken as 1 (Figure 5B). Thus, MS-275 was able to induce a concomitant cytotoxic action by potentially inducing a PARP1-mediated non-apoptotic cell death.

### 2.4. MS-275 Radiosensitizes FP-RMS Cells In Vitro

**Figure 6:**
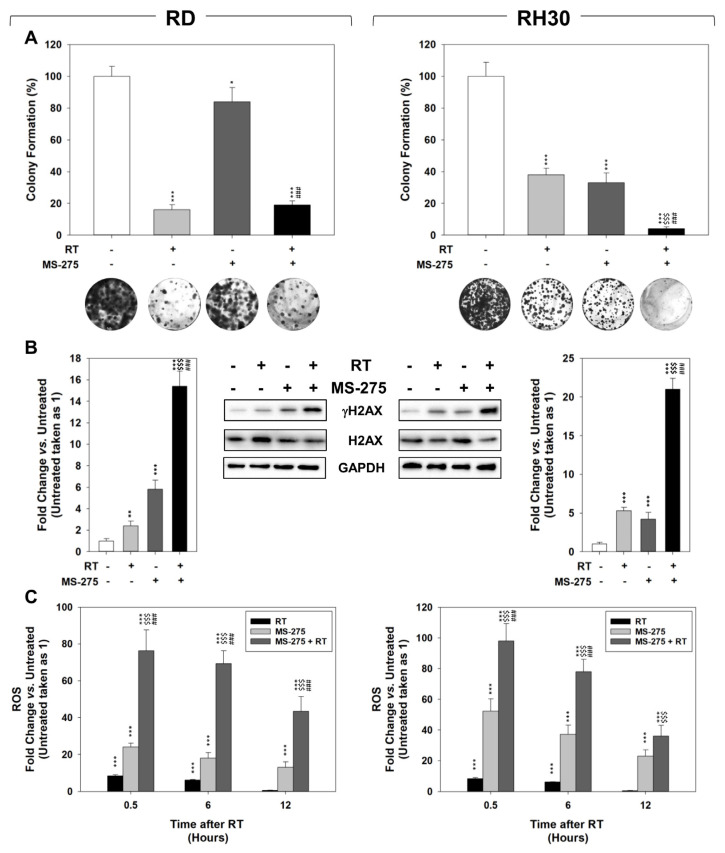
MS-275 radiosensitizes FP-RMS cell lines. (**A**) Colony formation assay of RD (Left) and RH30 (Right) treated with MS-275 IC_50_, RT alone or with the combination. Three hours after RT (4 Gy), cells were seeded at low concentrations for colony assays. Colony forming efficiency was calculated by crystal violet absorbance after 14 days of MS-275 treatment; RD (Left) and RH30 (Right). The lower panel shows representative pictures of colonies. Results represent the mean values ± SD of three independent experiments. (**B**) Cell lysates from RD (Left) and RH30 (Right) treated as in (**A**) were analyzed 6 h after irradiation by immunoblotting with specific antibodies for the indicated proteins. GAPDH expression was used as the loading control. Histograms of densitometric analysis (Left) represent the mean values of three independent experiments ± SD. (**C**) Mitochondrial super-oxide anion production of RD (Left) and RH30 (Right) treated as in (**A**) was assessed by MitoSox Red staining, half-hour (0.5), 6 or 12 h after RT. Statistical significance: * *p* ≤ 0.05, ** *p* ≤ 0.01, *** *p* ≤ 0.001 vs. Untreated cells; ^$$$^ *p* ≤ 0.001 vs. RT; ^###^ *p* ≤ 0.001 vs. MS-275.

The ability of MS-275 to sensitize to RT was assessed through colony formation assay performed on RMS cells pre-treated for 24 h with MS-275, then irradiated with a dose of 4 Gy and imaged 6 h later. As shown in Figure 6A, RT treatment alone inhibited the capability of both cell lines to form colonies (82.8 ± 4.7% in RD and 62.9 ± 3.9% in RH30 cells, respectively). In parallel, MS-275 as single agent reduced the colony formation ability of RD and RH30 cells by 18.2 ± 5.3% and 64.1 ± 4.9%, respectively and significantly potentiated the RT-induced toxicity in RH30 cells up to 87.2 ± 9.1% whilst it did not radiosensitize RD cells (Figure 6A). Then, the phosphorylation levels of H2AX (γ-H2AX), a specific molecular marker of DNA damage [46], were assessed in cell lysates at the end of the experiment, i.e., 6 h after irradiation, the time interval known to be sufficient for DNA repair in normal, but not cancerous, tissues [46]. Figure 6B shows that, compared to treatments alone, the presence of MS-275 markedly increases the ability of RT to upregulate γ-H2AX. Interestingly, MS-275 was able *per se* to increase the basal level of γ-H2AX in both RD and RH30 cells, confirming the ability of HDACi to induce DNA damage [47]. Then, we investigated whether MS-275 was capable to increase reactive oxygen species (ROS) production, known to be responsible for two-thirds of RT-induced DSBs [48]. To this purpose, RMS cells were pre-treated with MS-275 for 24 h and mitochondrial superoxide anion (O^2−^) production assessed 30 min (0.5), 6 and 12 h after irradiation. We noticed that ROS accumulation was triggered rapidly (0.5 h) by RT and MS-275 as single treatments in both RD and RH30 cells. However, while the MS-275-dependent ROS induction was maintained throughout the experiment, i.e., 12 h, the ROS levels due to RT returned to baseline 12 h later (Figure 6C).

**Figure 7:**
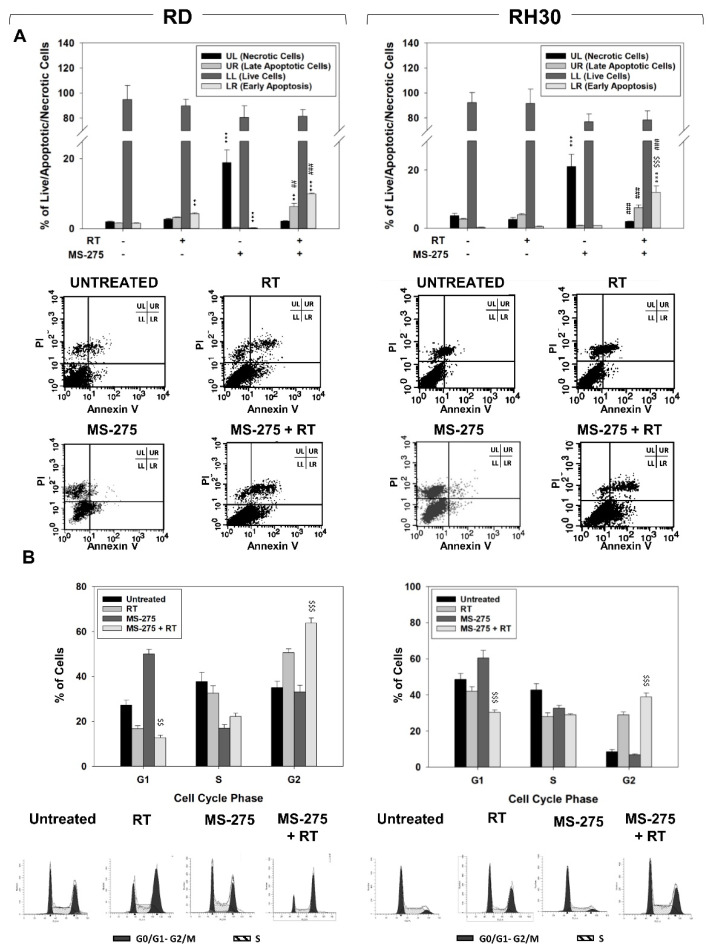
Pre-treating RMS with MS-275 promotes RT-induced apoptosis. (**A**) Annexin V analysis performed after 24 h of irradiation (4 Gy) on RD (Left) and RH30 (Right) cells untreated or treated with MS-275 IC_50_. Histograms (Up) show the percentage of necrotic and apoptotic cells representing the mean value of three independent experiments. Representative FACS plots (Down) of three different experiments. (**B**) FACS analysis performed on RD (left) and RH30 (right) treated as in (**A**). Histograms show the percentage of cells in the cell cycle phases and represent the mean value of four independent experiments. The lower panel shows a representative FACS plot of one of three independent experiments. Statistical significance: ** *p* ≤ 0.01, *** *p* ≤ 0.001 vs. Untreated cells; ^$$^ *p* ≤ 0.01, ^$$$^ *p* ≤ 0.001 vs. RT; ^##^ *p* ≤ 0.01, ^###^ *p* ≤ 0.001 vs. MS-275.

Pre-treating RMS cells with MS-275 further increased RT-induced ROS accumulation in both RD and RH30 cells and significantly impaired their ability to detoxify from ROS 12 h later, as occurred in cells subjected exclusively to RT (Figure 6C). The effects of MS-275 pre-treatment (24 h) in inducing RT cell death and modifying cell cycle distribution were investigated by Annexin V cell staining and flow cytometry, respectively, 24 h after irradiation. Compared to RT alone, pre-treating RMS with MS-275 increased the percentage of apoptotic cells (Figure 7A) and induced slight accumulation in the G2 phase of the cell cycle (Figure 7B). Altogether, these data indicate that the MS-275 predisposes both RD and RH30 to respond to radiation, even if it effectively radiosensitizes only RH30 cells. Furthermore, they also suggested a great ability of RD to repair radiation-induced damage and/or recover cell death.

### 2.5. In Vitro, MS-275 Counteracts the Ability of FP-RMS to Repair DNA Damage and Detoxify from ROS Accumulation Induced by RT

**Figure 8:**
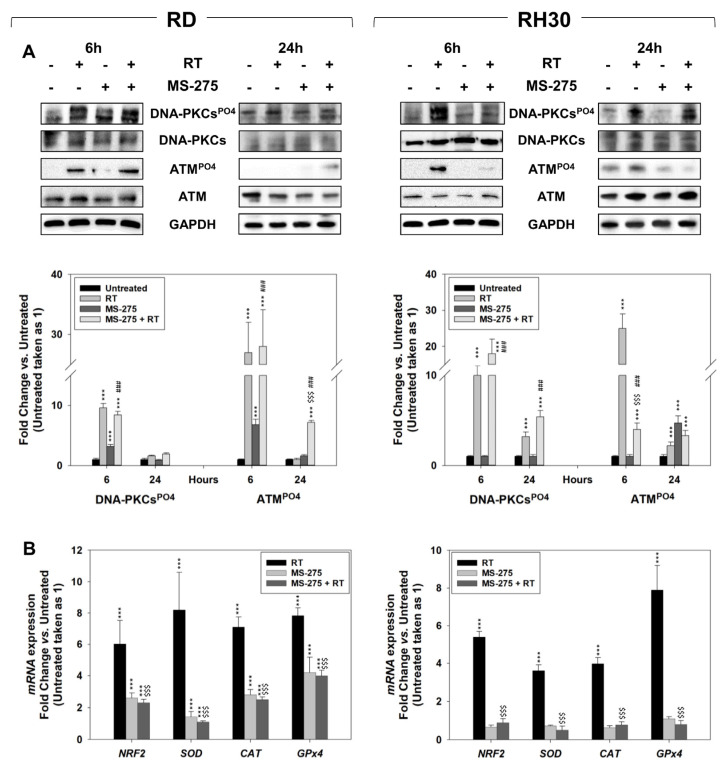
MS-275 counteracts the ability of RT to activate the HR-mediated DSBs repair pathway in RH30 and inhibits anti-oxidant molecular response in both RMS cell lines. (**A**) RD (Left) and RH30 (Right) were treated with MS-275 (IC_50_) and RT (4 Gy) alone or pre-treated (24 h) with MS-275 and then irradiated and values of three independent experiments ± SD. (**B**) Gene expression of antioxidant enzymes superoxide dismutase (*SOD-2*), catalase (*CAT*), glutathione peroxidase *(GPx)-4* and nuclear factor erythroid 2 p45-related factors (*NRF2*) were investigated by qRT-PCR, 12 h after RT in cells treated as in (**A**). The gene expression was reported as fold change vs untreated conditions reported equal to 1. Histograms are representative of three independent experiments performed in triplicate. Statistical significance: *** *p* ≤ 0.001 vs. Untreated cells; ^$$$^ *p* ≤ 0.001 vs. RT; ^###^ *p* ≤ 0.001 vs. MS-275.

The phosphorylation/activation status of DNA-PKCs and ATM signaling, respectively upstream of Non-Homologous End-Joining (NHEJ) and Homologous Recombination (HR) DSBs repair pathways, were investigated 6 h and 24 h after irradiation in RMS cells pre-treated or not for 24 h with MS-275. As shown in Figure 8A, MS-275 pre-treatment failed in counteracting the RT-induced activation of DNA-PKCs-dependent NHEJ pathway in both cell lines. Conversely, it was able to reduce the ability of RH30 cells to activate the ATM-dependent HR pathway (Figure 8A, RH30) whilst it failed in RD cells (Figure 8A, RD). The anti-oxidant cell response was investigated assessing the expression of the key master regulator *NRF2*, and of its target genes *SOD*, *CAT* and *GPx4* [49] by qRT-PCR, 12 h after irradiation in RMS cells pre-treated or not with MS-275. As shown in Figure 8B, the presence of MS-275 significantly restrained the mRNA accumulation of *NRF2*, *SOD*, *CAT* and *GPx4* induced by RT. The evidence herein collected suggests that MS-275 acts as a radiosensitizer in FP-RMS RH30 cells by preferentially impairing the activation of HR-DSBs repair and the anti-oxidant response.

### 2.6. MS-275 Radiosensitizes FP-RMS Cells In Vivo

**Figure 9:**
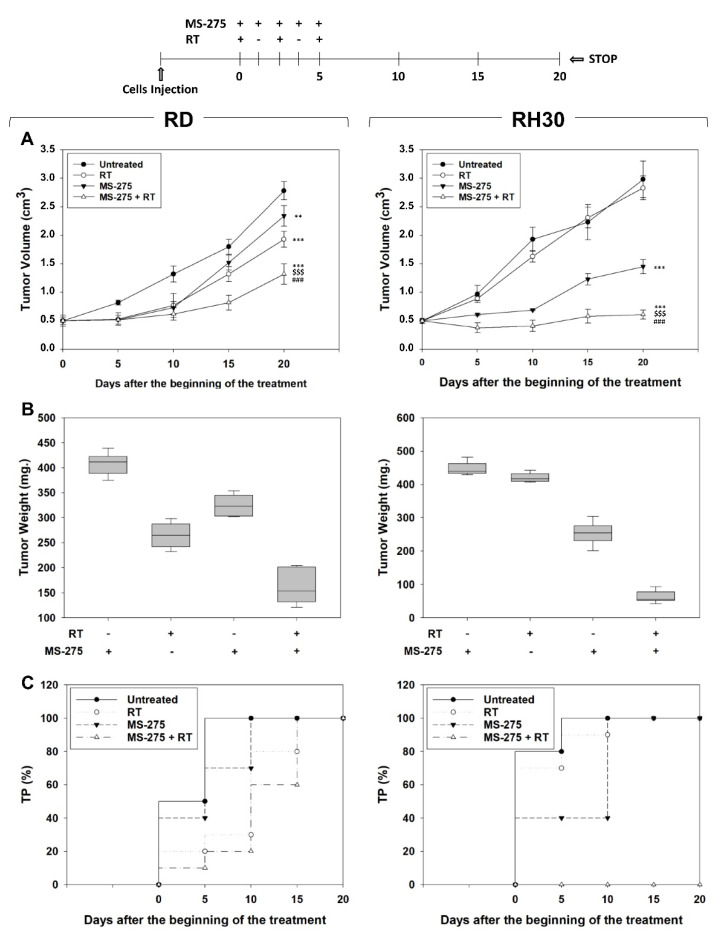
Effects of MS-275 combined or not with irradiation on in vivo tumor growth. The diagram above all the figures resumes how the experiment was performed. (**A**) Growth curve of tumor volumes from xenografted RD and RH30 cell lines, untreated, MS-275-treated, irradiated (RT), MS-275-pre-treated and irradiated (RT + MS-275). Tumor volumes were evaluated as described in methods and represent the mean ± SEM of 8 mice per group. The graphs show the sequential treatments of xenografted mice started when tumors reached a volume of approximately 0.5 cm^3^. Results represent the mean values ± SD. Statistical significance: ** *p* ≤ 0.01, *** *p* ≤ 0.001 vs. Untreated mice; ^$$$^ *p* ≤ 0.001 vs. RT-treated mice; ^###^ *p* ≤ 0.001 vs. MS-275-treated mice. (**B**) Tumor weights mice injected with RD (Left) and RH30 (Right) and treated with MS-275 and RT alone or in combination. (**C**) Kaplan–Meier estimates for rates of progression for untreated, MS-275, RT, or MS-275 + RT combination in RMS-derived tumors.

In vivo experiments were then performed by subcutaneously injecting (s.c.) RMS cells in nude mice. When the tumor volume reached ~0.5 cm^3^ (T0), mice were randomized into 4 groups of 8 animals each: vehicle, MS-275, RT, MS-275+RT. MS-275 as a single agent, 2.5 mg/kg [34], or vehicle (PBS) were administered intraperitoneally (i.p.) once daily for 5 consecutive days. Mice belonging to RT and MS-275+RT groups were irradiated with the dose of 2 Gy the 1st, 3rd and 5th day [37] one hour after receiving MS-275, for a total dose of 6 Gy. Tumor volumes were measured every 5 days for a period of 20 days after the starting of the treatment. Compared to single treatments, combining RT and MS-275 significantly improved the therapeutic efficiency resulting in 27.7 ± 7.9% volume reduction in RD and 75.4 ± 9.3% in RH30 xenografts compared to RT alone, and of 43.5 ± 8.2% in RD and 47.6 ± 7.2% in RH30 xenografts compared to MS-275 alone (Figure 9A). Remarkably, the co-treatment with MS-275 and RT completely prevented RH30 xenografts growth whilst RD xenografts progressively increased over the course of the experiment (Figure 9A). Accordingly, tumor weights of xenografts from mice co-treated with MS-275 and RT decreased significantly compared to those of untreated mice and single treatments (Figure 9B). Finally, the analysis of the number of mice showing tumor progression (TP), i.e., the doubling of the tumor volume, showed that in RD xenografted mice MS-275 and RT co-treatment slowed down the TP compared to MS-275 with no significant differences compared to RT alone, resulting in completed within the 15th day after the beginning of treatment (Figure 9C). Strikingly, no TP occurred in RH30 xenografted mice when co-treated (Figure 9C). Taken together, these findings highlight the ability of MS-275 to radiosensitize preferentially the FP-RMS subtype.

## 3. Discussion

In the present work, we investigated the potential of MS-275 (Entinostat), a potent class I and IV–selective HDACi [43], in sensitizing RMS cells to RT.

Accordingly to the already collected evidence [3,4,27], MS-275 induced growth arrest that washout experiments showed to be irreversible in FP-RMS RH30. Of note, we have recently shown that also the only class I HDACi FK228 (Romidepsin) induced growth arrest, but this was reversible for both RD and RH30 cells since the washout of the drug reverted the cytotoxicity [37]. Furthermore, the strong response of RH30 cells to MS-275 is in line with the effects of the drug on cell cycle distribution, which underwent a drastic modification with a high percentage of cells rapidly arrested in the G1 phase and cells that continue to die after washout.

Interestingly, although the FN-RMS did not undergo major changes in cell cycle distribution, both phenotypes exhibited similar changes at the molecular level. As a matter of fact, conversely to FK228 [37], MS-275: (i) down-regulated the expression of cell cycle positive regulators [50] cyclin-A, -B, -D1 and (ii) up-regulated the expression of the cyclin-dependent kinase inhibitors [50] p21 and p27 in both RMS cells subtypes; (iii) down-modulated the activation of MEKs/ERKs in RD and AKTs in RH30, which have been respectively shown to be among the key-master regulator signaling of FN- and FP-RMS [1].

One of the intriguing differences at the molecular level in RH30 cells, which could be related to the distinct response to MS-275 compared to FK228 [37], was the strong decrease of N-Myc levels. N-Myc is indeed one of the key core regulatory transcription factors (CR TFs) crucial for the maintenance of the FP-RMS tumorigenic phenotype, whose depletion determines the concomitant down-regulation of all the other CR TFs [51] and the death of cancer cells in vitro and in vivo [52].

On the two cell lines, MS-275 induced necrotic cell death potentially mediated by the activation of PARP1 [53,54] and similar to our previous study on FK228 [37]. However, despite a similar response to MS-275 in proliferation regulatory pathways in RD cells and RH30 cells, and the marked reduction of c-Myc in RD cells, an oncogene down-stream to the RAS pathway mutated in this cell line [55,56], MS-275 affected mildly the survival of RD cells, which still retained their ability to form growing colonies. This aspect deserves to be clarified in future studies to define whether the RAS pathway remains partially active under drug treatment.

Several mechanisms of chemoresistance to HDACi have been already described [17]. Herein, both surviving FP-RMS and FN-RMS cells up-regulated the expression of Bcl-2 and Bcl-xL, known to inhibit apoptotic [45] and PARP1-mediated-necrosis [57]. This mechanism, already described on RMS treated with other HDACi [36,37], while not sufficient to prevent FP-RMS RH30 cell death, could be strategic for FN-RMS RD cells to counteract the cytotoxic potential of HDAC inhibition and, therefore, could represent a potential further target for future investigation on HDACi-based combination approaches.

HDACi are known to trigger cell death also by inducing oxidative stress-mediated DSBs [58]. Accordingly, herein we found that MS-275 *per se* increased the accumulation of reactive oxygen species (ROS) in both cell lines and up-regulated the phosphorylation of H2AX (γ-H2AX), a marker for DSBs [46]. Since two-thirds of RT-induced DSBs depend on ROS accumulation [48] and HDACi-induced DNA damage cannot be easily repaired by transformed cells [57], we evaluated whether combining MS-275 with RT could be a good strategy to definitively kill RMS cells.

Herein, we found that, compared to single treatments, the combination of MS-275 and RT drastically impaired the clonogenic survival of FP-RMS but not FN-RMS cells even though significantly increased γ-H2AX in both. We supposed that this discrepancy could depend on the different ability of MS-275 to counteract the activation of molecular mechanisms of radioresistance in FP-RMS and FN-RMS. Cancer cells overcome RT principally by activating the DNA damage response (DDR) and the antioxidant responses [59]. Analyzing the phosphorylation/activation status of DNA-PKCs and ATM, respectively upstream of NHEJ and HR pathways of DDR [60], we found that MS-275 counteracted RT-induced activation of HR signaling in FP-RMS cells whilst did not affect DNA-PKCs activation in the two RMS subtypes. Therefore, the inability of MS-275 to radiosensitizes RMS cells seems to depend on its ability to counteract the full activation of both NHEJ and HR DSBs repair pathways induced by RT. On the other hand, RH30 radiosensitization appears to be related to the ability of MS-275 to prevent activation of the HR pathway, known to be the most important repair pathway and the last resort for DSBs repair [61]. In fact, HR has been shown to be critical to repair a wide variety of toxic lesions caused by many anticancer treatments and inactivation of the HR pathway may result in an increased sensitivity to anticancer treatments [61]. For example, the decreased expression of HR proteins correlates with a better response to DNA-damage-based therapies and their re-expression can restore cisplatin resistance [62,63]. Interestingly, in FP-RMS, HR activation resulted counteracted by MS-275 at least until six hours after irradiation, time interval commonly known to be sufficient for DNA repair in normal [64,65,66,67,68,69] but not in cancer cells [70]. Thus, six hours represents the minimum time between two consecutive radiotherapy fractions capable of the lowest toxicity [64,65,66,67,68,69] but also the time in which surviving neoplastic cells can be more easily killed by subsequent irradiation [71,72]. Thus the MS-275 would favor the radiosensitization to subsequent fractions of RT.

Of note, MS-275 drastically increased the ROS production and accumulation induced by RT, counteracted the ability of cells to detoxify from oxidative stress and enhancing their RT-dependent accumulation in the G2 phase of the cell cycle. Particularly, the presence of MS-275 restrained the ability of RMS cells to upregulate the expression of *NRF2* and of its related downstream targets *CAD*, *SOD* and *GPx4*, mediators of the antioxidant response, usually activated by ionizing radiation and responsible for radioresistance [73] as already reported in both FP-RMS and FN-RMS cells [74]. Notably, a similar effect has been described for FK228 [37]. However, while FK228 completely failed in RD and just restrained *NRF2* and *CAT* expression in RH30 cells [37], MS-275 counteracted the expression of the entire RT-induced molecular antioxidant axis in both cell lines. This finding suggests that FP-RMS cells have radioresistance mechanisms independent from the ROS detoxification mechanisms, an aspect that deserves further investigation.

The ability of MS-275 to radiosensitize FP-RMS was also confirmed in vivo by using RMS xenograft models. Pre-treating mice with MS-275 before RT completely prevent tumor growth as well as tumor progression in RH30 xenografted mice. This result could have a translational impact also considering that RH30 cells in vivo are completely unresponsive to RT and modestly respond to MS-275.

Notably, contrary to in vitro data, MS-275 radiosensitized FN-RMS in vivo even if the tumor masses only slowed their growth rate continuing to grow over time. A possible explanation for this discrepancy between in vitro and in vivo data could be given by the radiobiological concept of redistribution, which characterizes the functioning of the dose. It has been shown that cells arrested in G2 are more radiosensitive and consecutive fractions of RT, each of which can induce arrest in G2, can thus be progressively more and more effective [71]. Here, the pre-treatment with MS-275 increased the percentage of the cells in G2 making them more sensitive to the subsequent RT fractions. Further, other mechanisms could be involved in the in vivo response of FN-RMS cells, such as the ability of HDACi to inhibit angiogenesis [75] or tumor microenviroment [76]. Future experiments are needed to better understand the in vivo effects of MS-275 in FN-RMS in order to identify further strategies that definitively radiosensitize this tumor subtype. Finally, it cannot be excluded that the remarkable radiosensitizing effects of MS-275 especially on FP-RMS RH30 cells, could be, at least in part, related to the blockade of the only representative of class IV HDAC inhibited by the drug, i.e., HDAC11. This could be in line with the aberrant expression of this class of HDACs in this RMS subtype [77] and the effects of MS-275 on cell growth of another FP-RMS cell line [4]. Increasing evidence indicates HDAC11 as a rising star in epigenetics and potential therapeutic target for cancer treatment [77,78]. However, this hypothesis needs to be exploited in the future. Notably, deeper studies are needed to clarify the potential of combined HDACi and RT in cancer since it has been recently reported that adaptation mechanisms to radiation leading to radioresistance can be elicited by pan-HDAC inhibition [79]. In summary, we here showed that targeting class I and IV HDACs could be a potential therapeutic strategy to radiosensitize FP-RMS, the most aggressive type of RMS.

## 4. Materials and Methods

### 4.1. Cell Lines and Pharmacological Treatment

RD (ERMS) and RH30 (ARMS) human cell lines were purchased from American Type Culture Collection (Manassas, VA, USA). Cells were maintained as already described [80,81]. RD and RH30 were cultured respectively Dulbecco’s Modified Eagle’s and RPMI medium containing 10% Fetal Calf Serum (Hyclone, Logan, UT, USA) supplemented with glutamine and gentamycin (GIBCO-BRL Gaithersburg, MD, USA). GenePrint 10 System (Promega Corporation, Madison, WI, USA) was used to authenticate cell cultures by comparing the DNA profile of our cell cultures with those found in GenBank. The cells were dissociated using 0.25% trypsin and 0.02% EDTA solution and resuspended into a fresh medium once every 2–3 days. For the experiments, cells were seeded into 6-well tissue culture plates at a density of 8500 cells/cm^2^. One day after plating, cells were treated with Entinostat (MS-275) purchased from Selleckchem.com (Houston, TX, USA). Trypan blue (Thermofisher) exclusion was used to assess cell viability. Countess II Automated Cell Counter (ThermoFisher Scientific, Waltham, MA, USA) was used to assess the number of the cells. SigmaPlot (Systat Software, Inc, San Jose, CA, USA) software was used to calculate IC_50_ values. Histone Deacetylase (HDAC) Activity Assay Kit (Fluorometric) (ab156064) from abcam was used to test HDACs activity.

### 4.2. Cell Viability Assay

To assess cell viability, the day after plating, cells were exposed to MS-275 and at various times, all of the cells (adherent and non-adherent) were harvested. Viability was estimated as the proportion of cells that excluded 0.2% trypan blue (ThermoFisher Scientific, Waltham, MA). Countess II Automated Cell Counter (ThermoFisher Scientific, Waltham, MA) was used to assess the number of the cells. SigmaPlot (Systat Software, Inc, San Jose, CA, USA) software was used to calculate IC_50_ values.

### 4.3. Cell Cycle Analysis by Flow Cytometry

For the flow cytometry analysis, cells were harvested by trypsin-EDTA and washed; pellets were then resuspended in PBS in addition with 1% paraformaldehyde (final concentration of 0.5%) left at 4 °C for 1 hr. The fixed cells were then washed with PBS twice, resuspended in 0.3 mL of 50% FCS in PBS, in addition with 0.9 mL of 70% ethanol and left at 4 °C in the dark for no longer than 2 days before FACS analysis (BD FACSCalibur, BD Biosciences, Franklin Lakes, NJ, USA). ModFit LT 3.0 program (Verity Software House) was used to quantify flow cytometry data [82].

### 4.4. HDACs and PARP1 Activity Assays

To assess HDACs and PARP1 activity, the day after plating, cells were exposed to MS-275 and harvested at different times. Histone Deacetylase (HDAC) Activity Assay Kit (Fluorometric) (ab156064) from abcam was used to test HDACs activity and PARP1 Enzyme Activity Assay (17-10149) was from Sigma-Aldrich (St. Louis, MO, USA) to test PARP1 activity. Assays were performed accordingly with the manufacturer’s instructions.

### 4.5. Apoptosis Assay

The apoptotic cells were quantified (percentage) by using the Annexin V-CF Blue 7-AAD (ab214663, Abcam, Cambridge, UK). After treatments, cells were harvested at the indicated times, counted and Annexin-V labeling was performed according to the manufacturer’s instructions. Approximate fluorescence excitation maxima: 488 and 540 in nm. The stained cells were analyzed with a flow cytometer.

### 4.6. RNA Isolation and qRT-PCR

Total RNA was extracted by using TriPure Isolation Reagent (Euroclone, Italy). The concentration and quality of RNA were evaluated as already described [77]. QuantiTect Reverse Transcription Kit (Qiagen, Hilde, Germany) was used to perform the reverse transcription for target genes (*NRF2*, *SOD*, *CAT* and *GPx4*). A real-time PCR (qRT-PCR) was performed to analyze target genes [83]. For data analysis, the Ct values in each sample and the efficiencies of the primer set were calculated using LinReg Software and then converted into relative quantities (RQ) and normalized according to the Pfaffl model. Normalization was carried out using, as housekeeping genes, HPRT-1 for mRNA targets.

### 4.7. Mitochondrial Superoxide Anion (·O_2_−) Production Assessment

For mitochondrial ROS evaluation cells were with 5 μM MitoSox Red (Thermo Fisher Scientific, Italy, MI) in a pre-warmed medium for 15 min at 37 °C. After incubation, cells were washed twice, first with pre-warmed complete medium and then with PBS, trypsinized and centrifuged at 2500 rpm, the pellet was resuspended in PBS with 3% FBS for immediate analysis in a BD FACS (BD FACS Calibur, BD Biosciences, Franklin Lakes, NJ, USA).

### 4.8. Radiation Exposure and Clonogenic Assay

Radiation was delivered at room temperature using an x-6 MV photon linear accelerator, as previously described [84]. The total single dose of 4 Gy was delivered with a dose rate of 2 Gy/min using a source-to-surface distance (SSD) of 100 cm. A plate of Perspex thick 1.2 cm was positioned below the cell culture flasks in order to compensate for the build-up effect. Tumor cells were then irradiated placing the gantry angle at 180°. Non-irradiated controls were handled identically to the irradiated cells with the exception of the radiation exposure. The absorbed dose was measured using a Duplex dosimeter (PTW). For clonogenic survival assay, exponentially growing RD and RH30 cells in 25-cm^2^ flasks were harvested by exposure to trypsin and counted. They were diluted serially to appropriate densities and plated in triplicate in 6 multi-well plates with 2 mL of complete medium/each well in the presence or absence of MS-275 or vehicle for 24 h (final DMSO concentration of 0.1%; we confirmed that this DMSO concentration did not affect the proliferation of RD and RH30 cell lines). After incubation for 24 h, the cells were exposed at room temperature to various doses of radiation as already described. The cells were then washed with PBS, cultured in a drug-free medium for 14 days, fixed with methanol:acetic acid (10:1, *v/v*), and stained with crystal violet. Colonies containing >50 cells were counted. The plating efficiency (PE) was defined as the number of colonies observed/the number of cells plated; the surviving fraction (SF) was calculated as follows: colonies counted/cells seeded X (PE/100) [84].

### 4.9. Protein Extraction and Western Blot

Cells were lysed in 2% SDS containing 2 mM phenyl-methyl sulphonyl fluoride (PMSF) (Sigma-Aldrich (St. Louis, MO, USA), 10 μg/mL antipain, leupeptin and trypsin inhibitor, 10 mM sodium fluoride and 1 mM sodium orthovanadate (all from Sigma-Aldrich (St. Louis, MO, USA)) and sonicated for 30 s. Protein concentration was estimated by BCA assay and equal amounts were separated on SDS-PAGE. The proteins were transferred to a nitrocellulose membrane (ThermoFisher Scientific, Waltham, MA, USA) by electroblotting. The balance of total protein levels was confirmed by staining the membranes with Ponceau S (Sigma-Aldrich (St. Louis, MO, USA). Membranes were blocked for 1 h in 5% non-fat milk in Tris-buffered saline and Tween-20 (TBS-T) and then incubated at 4° C overnight with primary antibodies [85]. The primary antibodies used were: p21^WAF1^ (C-19), p27^KIP1^ (F-8), Cyclin A (BF683), Cyclin D1 (M-20), Cyclin B1 (H-20), myelocytomatosis virus oncogene cellular homolog (c-Myc) (9E10), N-Myc (B.8.4.B), phosphorylated extracellular signal-regulated kinase 1/2 (ERK1/2PO4) (E-4), extracellular signal-regulated kinase (ERK1/2) (C-14, positive also for ERK1), H2A histone family member X (H2AX) (C-20), phospho-ATM (10H11.E12, Ser1981), ATM (H-248), DNA-PKCs (E-6), GAPDH (0411) and α-Tubulin (TU-02) by Santa Cruz Biotechnology (Dallas, TX, USA); phosphorylated H2A histone family member X (γH2AX) (Ser139) (2577), Bcl2 (D55G8) (4223), BCL-xl (54H6) (2764) by Cell Signaling Technology (Danvers, MA, USA); phospho-DNA-PKCs (Thr2609) (10B1) by AbCam (Cambridge, UK). Appropriate horseradish peroxidase (HRP)-conjugated secondary antibodies (Santa Cruz Biotechnology (Dallas, TX, USA)) were used for 1 h at room temperature. Quantification of western blot data was performed by using ChemiDoc MP (Bio-Rad) imager.

### 4.10. Animal Research Ethics Statement and In Vivo Xenograft Experiments

The recommendations of the European Community (EC) guidelines (2010/63/UE and DL 26/2014 for the use of laboratory animals) and the University guidelines (University of L’Aquila, Board Regulations on the use of laboratory animals) were used to perform in vivo experiments. Before any invasive manipulation, mice were anesthetized with a mixture of ketamine (25 mg/mL)/xylazine (5 mg/mL). For xenotransplants exponentially growing RD or RH30 cells were detached by trypsin-EDTA, washed twice in PBS, and resuspended in saline solution at cell densities of 1 × 106/200 μL. Xenotransplants were done in 45-day-old female nude CD1 mice from Charles River Laboratories Italia, SRL (Calco, Italy), by s.c. injection in the leg using a 21-gauge needle on a tuberculin syringe. Treatments started when tumors reached a volume of 0.5 cm^3^ [36,37]. MS-275 was administered 2.5 mg/kg/day [34] by intraperitoneal (i.p.) injection for 5 days/week for 1 week. The first daily dose of MS-275 was given one hour before RT. Mice were irradiated at room temperature using an Elekta 6-MV photon linear accelerator. Three fractions of 2 Gy were delivered every other day, the 1st, 3rd and 5th day, for a total dose of 6 Gy. A dose rate of 1.5 Gy/min will be used with a source-to-surface distance (SSD) of 100 cm. Prior to irradiation mice were anesthetized and were protected from off-target radiation by a 3 mm lead shield. Before tumor inoculation mice were randomly assigned to 4 experimental groups. Each group was composed of 8 mice. One control group received intraperitoneal (i.p.) injection of 200 μL carrier solution; one group received i.p. injection of 200 μL MS-275 solution at the dose of 2.5 mg/kg/day; one group received RT (3 fractions of 2 Gy delivered every other day to a total dose of 6 Gy); one group received 200 μL MS-275 solution at the dose of 2.5 mg/kg/day coupled with RT (3 fractions of 2 Gy delivered every other day to a total dose of 6 Gy). During treatment, mice with significant body weight loss approaching (10–15%) were euthanized early per protocol.

### 4.11. Evaluation of Treatment Response In Vivo

The effects on tumor growth of different treatments were evaluated as follows: (1) measuring tumor volume during and at the end of the experiment. Tumor volume was assessed every 4 days measurement with a Vernier caliper (length × width). The volume of the tumor was expressed in mm3 according to the formula 4/3π r3, measuring tumor weight at the end of the experiment and defining tumor progression (TP), the doubling of the tumor volume.

### 4.12. Statistical Analysis and Data Analysis

Three independent experiments, each performed in triplicate, were performed and the results were expressed as the mean ± SD. Data normal distribution was confirmed by Shapiro–Wilk, D’Agostino and Pearson and Kolmogorov–Smirnov tests. Real-time PCR experiments were evaluated by one-way (ANOVA) with a Tukey’s post hoc test using 2^−ΔΔCt^ values for each sample. Flow cytometry data were analyzed by ANOVA with a Bonferroni post hoc test. All analyses were performed using the SAS System (SAS Institute Inc., Cary, NC, USA) and GraphPad Prism 6.1.

## Data Availability

Not applicable.
